# First isolation and genetic characterization of pseudocowpox virus from cattle in Japan

**DOI:** 10.1186/s12985-017-0840-3

**Published:** 2017-09-06

**Authors:** Akifumi Ohtani, Akihiro Yokoyama, Hisato Narushige, Yasuo Inoshima

**Affiliations:** 1 Yamaguchi Chubu Livestock Hygiene Service Center, 671-5 Kagawa, Yamaguchi, Yamaguchi, 754–0897 Japan; 2 Laboratory of Food and Environmental Hygiene, Cooperative Department of Veterinary Medicine, Gifu University, 1-1 Yanagido, Gifu, Gifu, 501-1193 Japan; 3 The United Graduate School of Veterinary Sciences, Gifu University, 1-1 Yanagido, Gifu, Gifu, 501-1193 Japan; 4 Education and Research Center for Food Animal Health, Gifu University (GeFAH), 1-1 Yanagido, Gifu, Gifu, 501-1193 Japan

**Keywords:** Pseudocowpox virus, Cattle, Oral lesions, Isolation, *B2L* gene

## Abstract

**Background:**

Pseudocowpox virus (PCPV) infects cattle worldwide with zoonotic potential but has not been isolated in Japan. Thus, the epidemiological status of PCPV infection in cattle is undetermined.

**Results:**

In May 2016, a cattle in a farm in Yamaguchi Prefecture showed white vesicles and hyperemia in the mucosa under the tongue surface, but not on the teats and coronary cushions. A parapoxvirus was isolated from the oral lesion swab and was genetically characterized based on the full-length sequence of *B2L* gene encoding viral envelope. Phylogenetic analysis showed that the isolated virus was classified into PCPV.

**Conclusion:**

This case indicates its potential spread in Japan. This is the first report of isolation of PCPV in Japan.

**Electronic supplementary material:**

The online version of this article (10.1186/s12985-017-0840-3) contains supplementary material, which is available to authorized users.

## Background

Pseudocowpox virus (PCPV) is a member of the genus *Parapoxvirus* in the family *Poxviridae*, which includes bovine papular stomatitis virus (BPSV) and orf virus (ORFV) [1]. Parapoxviruses are commonly known as causative agents of dermal diseases in ruminants worldwide, leading to papular stomatitis and contagious pustular dermatitis, especially in the regions of the lips, nostrils, oral mucosa, and teats. The importance of PCPV is increasingly recognized, primarily because of economic losses to farmers in connection with disease outbreaks and because of their zoonotic potential [2].

In Japan, although serological surveys have revealed that seroprevalence of parapoxvirus is very high in cattle and sheep [3, 4] and multiple BPSVs have been isolated [5], no PCPV has yet been isolated; thus, the epidemiological status of PCPV infection in cattle is undetermined.

We here report the first case of the isolation of PCPV in Japan. We determined the full-length sequence of the *B2L* gene encoding viral envelope of this isolate, and evaluated its phylogenetic relation to known members of this virus group.

## Methods

### Clinical and epidemiological investigations

**Fig. 1:**
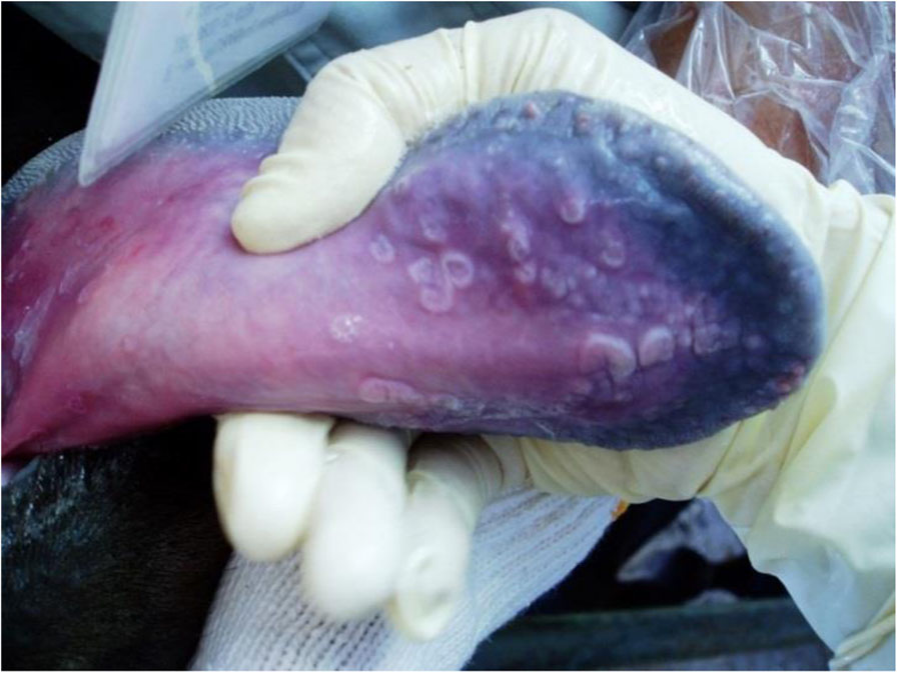
Clinical presentation of an affected cattle with white vesicles in the mucosa under the tongue surface

In May 2016, a breeding cow (Japanese Black, female, 13-month old) in a farm in Yamaguchi Prefecture, in the western part of Japan showed anorexia, mild fever, frothy salivation, and hyperemia in the mucosa under the tongue surface. No lesions were observed on the teats or coronary cushions. A few days after the onset of clinical signs, the cattle showed white vesicles on the tongue surface (Fig. 1). These signs were convalescent in about 1 week. No other cattle in the herd showed clinical signs.

### Sample collection

An oral swab sample was collected from the mucosal lesions of the affected cattle and was homogenized with Eagle’s minimum essential medium (MEM). The sample was centrifuged at 800×*g* for 10 min at 4 °C. The supernatant was filtered through a 450-nm membrane (Merck Millipore, Cork, Ireland) and used for virus isolation and DNA extraction.

### Virus isolation

Virus isolation was performed through inoculation in primary bovine testis (BT) cells; two cell lines, hamster lung (HmLu-1) and Madin-Darby bovine kidney (MDBK) cells were also tested for comparison. The cells were grown in Eagle’s MEM with kanamycin (Nissui Pharmaceutical, Tokyo, Japan) supplemented with 0.295% tryptose phosphate broth (TPB), 0.015% sodium bicarbonate, 0.03% l-glutamine, and 5–10% fetal bovine serum at 37 °C. The cells were cultured in rolling tubes and 24-well plates, washed with maintenance medium (MEM containing 0.295% TPB, 0.015% sodium bicarbonate, 0.03% l-glutamine, and 0.1% bovine serum albumin), and inoculated with 0.1 ml of the processed samples. After adsorption for 60 min at 37 °C, the inocula were replaced with 0.5 ml of maintenance medium. The rolling tube-cultured and 24-well plates-cultured cells were incubated by rotary cultures and stationary cultures, respectively, and observed daily for cytopathic effects (CPE) for at least 7 days. Cultures without CPE were passaged twice in a blinded manner.

### Genetic analysis

DNA was extracted both from the oral swab sample and from the BT cells showing a CPE using magLEAD 12gC (Precision System Science, Chiba, Japan). Polymerase chain reaction (PCR) amplifications were carried out with TaKaRa Ex Taq Hot Start Version (TaKaRa Bio, Shiga, Japan) using a SimpliAmp Thermal Cycler (Thermo Fisher Scientific, Kanagawa, Japan) for detection of the full-length (1137 bp) and partial-length (554 bp) *B2L* gene encoding envelope of parapoxvirus with the primer sets OVB2LF1/OVB2LR1 [6] and PPV1/PPV4 [5], respectively. PCR products were purified using NucleoSpin Gel and PCR Clean-up (Macherey-Nagel, Düren, Germany), and the nucleotide sequence was determined by direct sequencing using a BigDye Terminator Cycle Sequencing Kit v3.1 (Applied Biosystems, Austin, TX, U.S.A.). Sequence data were aligned using the ClustalW method [7]. Phylogenetic analysis was performed using MEGA 6 software [8]. Phylogenetic trees were constructed using maximum-likelihood methods, and the reliability of the branches was evaluated by bootstrapping with 1000 replicates. Nucleotide and deduced amino acid sequences were compared with those of available corresponding parapoxviruses (Table 1). Bovine viral diarrhea virus [9], epizootic hemorrhagic disease virus [10], bluetongue virus [10], ovine herpesvirus 2 [11], and bovine herpesvirus 1 [12], were not detected by PCR using specific primers for detection of each viruses (data not shown).

**Table 1:** Nucleotide and deduced amino acid sequence identities (%) of the full-length *B2L* gene

Virus^a^	Strain	Host	Nucleotide	Amino acid	Accession number
PCPV	YG2828	Cattle	–	–	LC230119
	VR634	Cattle	98.6	99.2	GQ329670
	TQ	Cattle	98.2	98.7	AY424972
	F07.798R	Reindeer	98.4	98.9	JF773692
	F07.801R	Reindeer	98.4	98.9	JF773693
	F05.990C	Cattle	98.6	98.7	JF773694
	F10.3081C	Cattle	98.6	98.7	JF773695
	F00.120R	Reindeer	98.4	98.9	GQ329669
	Arero/05/2013	Camel	97.2	97.6	KU645549
	Arero/04/2013	Camel	97.0	97.1	KU645548
	Arero/02/2014	Camel	97.2	97.6	KU645546
	Hordha/01/2011	Camel	97.0	97.1	KU645563
ORFV	NZ2	Sheep	94.1	95.0	DQ184476
	F07.808R	Reindeer	93.7	93.9	JF773698
	SD/DY	Sheep	93.9	93.7	JQ904794
	Adet/O03/2012	Sheep	94.0	95.0	KT438515
BPSV	RS	Cattle	85.8	84.4	AY424973
	BV-TX09c1	Cattle	85.8	84.7	KM875472
	BV-TX09c5	Cattle	85.8	84.7	KM875471
	BV-AR02	Cattle	86.1	84.7	AY386265

^a^PCPV, pseudocowpox virus; ORFV, orf virus; BPSV, bovine papular stomatitis virus

## Results

**Fig. 2:**
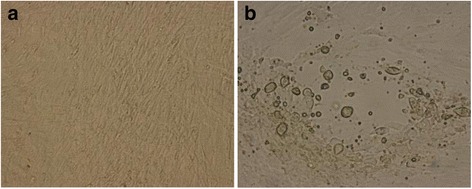
Cytopathic effect observed in BT cells at passage 3. The cells were tested at day 3 after inoculation. Non-infected control (**a**) and infected (**b**) cells are shown

At the third passage, a distinct CPE was observed from 2 to 3 days after inoculation in the rolling tube-cultured BT cells, characterized by a rounded morphology and cell detachment (Fig. 2). We designated the isolate as strain YG2828. However, at the third passage, no CPE appeared in the other cell lines or in stationary cultures. For histological observations, confluent monolayers of BT cells in the chamber slide system were inoculated with the isolate. Twenty-four hours after inoculation, the BT cells were fixed with acetone and stained with hematoxylin-eosin, which revealed pyknosis, and eosinophilic and basophilic cytoplasmic inclusion bodies (data not shown).

**Fig. 3:**
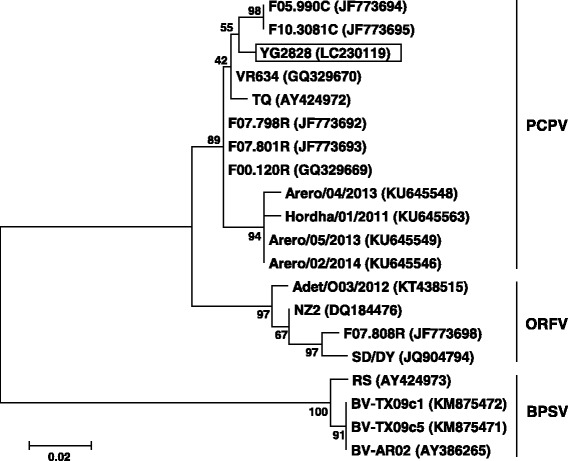
Phylogenetic tree of parapoxviruses based on the deduced amino acid sequence of the full-length *B2L* gene (378 amino acids). The percentage bootstrap values calculated from 1000 replications are indicated above the internal nodes

Fragments of expected size were amplified by PCR using both primer sets. Neither deletions nor insertions in the nucleotide sequence of the YG2828 strain were found (Additional file 1: Figure S1). Based on the nucleotide/amino acid identities and phylogenetic analysis of the full-length *B2L* gene, the YG2828 strain was classified as PCPV (Fig. 3). The nucleotide identities against published parapoxviruses ranged from 85.8 to 98.6% (Table 1), and showed the highest identity (98.6%) to three PCPV strains: F05.990C and F10.3081C isolated from cattle in 2005 and 2010 in Finland, respectively, and VR634 isolated from a human in the USA in 1963 with “milker’s nodules” on the hands. The deduced amino acid identities ranged from 84.4 to 99.2%, and showed the highest identity (99.2%) to the VR634 strain (Table 1), even though these strains were isolated independently, chronologically, and geographically.

## Discussion

As noted above, seroprevalence of parapoxvirus is very high in cattle in Japan and multiple BPSVs have been isolated [5], but no PCPV has yet been isolated. In this study, a PCPV was firstly isolated in Japan by rotary cultures, but not stationary cultures. Similarly, Mavromoustakis et al. [13] reported that significantly (*P* < 0.01) less herpes simplex virus was produced in stationary than in rotary cultures. Although the procedures of rotary cultures are more burdensome than those of static cultures, we suggest that rolling of inoculated cultures should be conventionally applied in clinical virology laboratories to aid in the isolation of PCPV. In the affected cattle in this study, there was no evidence of infection on the teats and udder, which are the more common lesion sites of pseudocowpox infection. The classification of parapoxviruses was formerly based on the natural host range, clinical signs, and serology [14], however this does not always reflect the classification revealed by molecular analysis [5] as evidenced by the present study.

Previously, there has only been one report describing the PCR detection of PCPV DNA in Japan, in which PCPV DNA was detected from oral lesions in a calf in Iwate Prefecture, in the northern part of Japan, but virus isolation was unsuccessful [15]. Notably, the partial-length sequence of the *B2L* gene determined from the PCR product (accession no. AB921003) was identical to that of the present strain YG2828 (data not shown). Thus, our results confirmed that PCPV can be isolated from atypical sites besides the teats and udder, and suggest that YG2828-like PCPV may cause oral lesions in cattle. Moreover, since parapoxviruses cross-react antigenically and two similar strains infected cattle in different locations in separate years, YG2828-like PCPV might be spreading among the cattle population in Japan. It is known that cattle are frequently infected with parapoxvirus subclinically [15] and PCPV has zoonotic potential [2]. Therefore, we recommend to wear gloves for people with regular exposure to cattle mucosa.

## Conclusion

A PCPV was firstly isolated in Japan from the oral lesion swab of cattle showing white vesicles and hyperemia in the mucosa under the tongue surface, but not on the teats and coronary cushions, by rotary cultures. Genetic characterization based on the full-length sequence of *B2L* gene revealed that the isolated virus was genetically close to strains isolated from cattle in the USA and Finland. PCPV is responsible for significant economic losses in the cattle production. Further virological and epidemiological studies to characterize this strain and the possibility of its spread in Japan are highly required.

## Additional file

Additional file 1: Figure S1. Alignment of the deduced amino acid sequences of the full-length *B2L* gene. Amino acids identical to the pseudocowpox virus strain YG2828 at given positions are represented by dots. (PPTX 73 kb)

## Abbreviations

BPSV: Bovine papular stomatitis virus
BT: Bovine testis
CPE: Cytopathic effects
HmLu-1: Hamster lung
MDBK: Madin-Darby bovine kidney
MEM: Minimum essential medium
ORFV: Orf virus
PCPV: Pseudocowpox virus
PCR: Polymerase chain reaction
TPB: Tryptose phosphate broth

## References

[1] Knowles DP. (2011) Poxviridae. In: Maclachlan NJ, Dubovi FJ, editors. Fenner’s Veterinary virology. 4th ed. London: Academic Press; 2011. p. 151–65.

[2] Friederichs S, Krebs S, Blum H, Wolf E, Lang H, von Buttlar H, Büttner M (2014). Comparative and retrospective molecular analysis of parapoxvirus (PPV) isolates. Virus Res.

[3] Kuroda Y, Yoshida M, Shibahara T, Matsui T, Nakane T, Hara H, Inoshima Y, Sentsui H (1999). An epidemic of parapoxvirus infection among cattle : isolation and antibody survey. J Vet Med Sci.

[4] Sentsui H, Inoshima Y, Minami A, Yamamoto Y, Murakami K, Shimizu S (2000). Survey on antibody against parapoxvirus among cattle in Japan. Microbiol Immunol.

[5] Inoshima Y, Murakami K, Yokoyama T, Sentsui H (2001). Genetic heterogeneity among parapoxviruses isolated from sheep, cattle and Japanese serows (*Capricornis crispus*). J Gen Virol.

[6] Hosamani M, Bhanuprakash V, Scagliarini A, Singh RK (2006). Comparative sequence analysis of major envelope protein gene (B2L) of Indian orf viruses isolated from sheep and goats. Vet Microbiol.

[7] Thompson JD, Higgins DG, Gibson TJ (1994). CLUSTAL W: improving the sensitivity of progressive multiple sequence alignment through sequence weighting, position-specific gap penalties and weight matrix choice. Nucleic Acids Res.

[8] Tamura K, Stecher G, Peterson D, Filipski A, Kumar S (2013). MEGA6: Molecular evolutionary genetics analysis version 6.0. Mol Biol Evol.

[9] Vilček Š, Herring AJ, Herring JA, Nettleton PF, Lowings JP, Paton DJ (1994). Pestiviruses isolated from pigs, cattle and sheep can be allocated into at least three genogroups using polymerase chain reaction and restriction endonuclease analysis. Arch Virol.

[10] Ohashi S, Yoshida K, Yanase T, Kato T, Tsuda T (2004). Simultaneous detection of bovine arboviruses using single-tube multiplex reverse transcription-polymerase chain reaction. J Virol Methods.

[11] Baxter SIF, Pow I, Bridgen A, Reid HW (1993). PCR detection of the sheep-associated agent of malignant catarrhal fever. Arch Virol.

[12] Rocha MA, Barbosa EF, Guimarães SEF, Dias Neto E, Gouveia AMG (1998). A high sensitivity-nested PCR assay for BHV-1 detection in semen of naturally infected bulls. Vet Microbiol.

[13] Mavromoustakis CT, Witiak DT, Hughes JH (1988). Effect of high-speed rolling on herpes simplex virus detection and replication. J Clin Microbiol.

[14] Robinson AJ, Lyttle DJ, Binns M, Smith GL (1992). Parapoxviruses : their biology and potential as recombinant vaccines. Recombinant Poxviruses.

[15] Yaegashi G, Sasaki I, Chiba S, Murakami K (2013). Molecular analysis of parapoxvirus detected in eight calves in Japan. J Vet Med Sci.

